# Monolayer graphene/SiC Schottky barrier diodes with improved barrier height uniformity as a sensing platform for the detection of heavy metals

**DOI:** 10.3762/bjnano.7.173

**Published:** 2016-11-22

**Authors:** Ivan Shtepliuk, Jens Eriksson, Volodymyr Khranovskyy, Tihomir Iakimov, Anita Lloyd Spetz, Rositsa Yakimova

**Affiliations:** 1 Department of Physics, Chemistry and Biology, Linköping University, SE-58183, Linköping, Sweden

**Keywords:** barrier height, graphene, heavy metals, Schottky diode, sensing platform, SiC

## Abstract

A vertical diode structure comprising homogeneous monolayer epitaxial graphene on silicon carbide is fabricated by thermal decomposition of a Si-face 4H-SiC wafer in argon atmosphere. Current–voltage characteristics of the graphene/SiC Schottky junction were analyzed by applying the thermionic-emission theory. Extracted values of the Schottky barrier height and the ideality factor are found to be 0.4879 ± 0.013 eV and 1.01803 ± 0.0049, respectively. Deviations of these parameters from average values are smaller than those of previously observed literature data, thereby implying uniformity of the Schottky barrier height over the whole diode area, a stable rectifying behaviour and a good quality of ohmic palladium–graphene contacts. Keeping in mind the strong sensitivity of graphene to analytes we propose the possibility to use the graphene/SiC Schottky diode as a sensing platform for the recognition of toxic heavy metals. Using density functional theory (DFT) calculations we gain insight into the nature of the interaction of cadmium, mercury and lead with graphene as well as estimate the work function and the Schottky barrier height of the graphene/SiC structure before and after applying heavy metals to the sensing material. A shift of the *I*–*V* characteristics of the graphene/SiC-based sensor has been proposed as an indicator of presence of the heavy metals. Since the calculations suggested the strongest charge transfer between Pb and graphene, the proposed sensing platform was characterized by good selectivity towards lead atoms and slight interferences from cadmium and mercury. The dependence of the sensitivity parameters on the concentration of Cd, Hg and Pb is studied and discussed.

## Introduction

As a result of reckless and uncontrollable human activity many dangerous non-biodegradable substances are released into the atmosphere and water sources [1]. Among them, Cd, Hg and Pb are highly toxic heavy metals (HMs), which may be responsible for the development of intractable diseases, thereby creating huge unavoidable problems for living creatures [2]. Therefore, the pollution induced by hazardous heavy metals is a great challenge for global sustainability. In this respect, it is of vital importance to propose and comprehensively investigate a real-time sensing platform using an eco-friendly material, with high chemical activity and tunable intrinsic electronic properties. It is also important to note that the most popular approaches to detect heavy metals are mainly based on complicated chromatography principles [3], photometric methods [4], atomic absorption spectroscopy [5], mass spectrometry [6] and total reflection X-Ray fluorimetry [7]. These techniques provide high limits of detection, but require very expensive analytical instruments developed for the utilization in laboratories. Thus, in many cases they are not portable and cannot be used for online monitoring. Nevertheless, the Swedish company Envic-Sense (http://envic-sense.com/) has patented a technology based on a quartz crystal microbalance (QCM) to detect toxic heavy metals such as cadmium, mercury, arsenic, and lead in water and soil samples. The working principle of such detectors is based on the dependence of the frequency of a quartz crystal resonator on the concentration of the foreign substances to be detected.

To get a full picture of the state of the art describing all meaningful existing approaches to detect heavy metals, we also need to mention anode stripping voltammetry (ASV). ASV uses carbon, mercury and bismuth electrodes (and others) and is a well-known method for the identification of heavy metals [8–13]. Nevertheless, this technique has some disadvantages, which are related to (1) the failure to detect Hg, Ag, Au and metals not forming amalgams (in the case of using a mercury electrode) [8]; (2) extensive analysis time and the required skills of the operating staff; (3) the observation of interferences and additional ASV peak potentials originating from background contamination and overlapping potentials of the involved HMs [8–10]; (4) the toxicity of conventional mercury drop electrodes [11]; (5) the difficulty to determine the concentration of individual heavy metals in simultaneous presence of other heavy metals [8]; 6) passivation of the electrodes due to adsorption of different non-metallic substances [9]; (7) poor reproducibility due to the formation of intermetallic compounds; (8) hydrolysis at the electrode [12]. Furthermore, an additional drawback of this technique is that most of the metallic and carbon-based electrodes are characterized by a narrow negative potential window, which is not sufficient to detect the metals with high values of the electronegativity [12]. To solve these problems and to improve the sensing characteristics one need to chemically modify the surface of the electrodes (surface functionalization) [8,13], which will complicate the fabrication of the sensors and reduces the reproducibility.

Taking the above discussion into account, one can conclude that the need to create accurate and real-time analytical instruments for detection of heavy metals still exists. A solution to this problem can be achieved not only through the improvement of the existing techniques, but also through the development of new approaches. One of the most promising candidates for the development of real-time detectors for heavy metals is graphene [14]. Due to its large surface area (2600 m^2^/g) [15], high chemical activity [16] and exceptionally high signal-to-noise ratio [17], graphene provides a rich platform for surface chemistry and the desirable conditions for detection of heavy metals because of the strong sensitivity of its electronic properties to a change in concentrations of surface functional groups and adsorbates.

However, sensors based on reduced graphene oxide are only well investigated in terms of determination of the concentration limit of heavy metals and improving the response time [18–26]. In particular, it was previously reported that functionalized graphene oxide sheets on Au templates can effectively detect lead and mercury ions with improved electrochemical performance [18]. The possibility of using field effect transistors (FET) based on thermally reduced graphene oxide decorated with functionalized gold nanoparticles and DNA for detecting mercury ions in aqueous solution was also demonstrated [19,22]. It has to be pointed out that graphene loses part of its exotic properties after oxidation, thereby degrading its intrinsically high sensing capability. Furthermore, according to literature, graphene oxide is more toxic than pristine graphene [27], has a lower carrier mobility [28], higher thermal noise and a natural tendency to agglomerate [29]. In addition, because of the high material inhomogeneity and small domain sizes, it is complicated to fabricate sensing devices in which the active area of the device comprises only one uniform and continuous monolayer (ML). To date, no sensors for the detection of heavy metals based on pristine graphene or even epitaxial graphene have been tested.

In many cases FET devices are considered as effective sensing platforms for heavy metals [30–31]. The main drawback of the FET-based sensors is the complex fabrication, followed by a necessity to grow the layers of high-*k* gate dielectrics. These additional steps can result in the formation of unexpected and uncontrollable interface states, deteriorating the output characteristics of the devices and their sensitivity. A simpler solution is to use Schottky diode sensors, which can be grown more easily, have no gate insulator and a high sensitivity in the reverse and forward diode regimes.

During the last decade the thermal decomposition of silicon carbide (SiC) in argon atmosphere was shown to be a reliable and effective approach for the formation of homogenous epitaxial graphene layers with controllable thickness [32–33]. Combining these two materials (graphene and SiC) and considering a strong ability of graphene to interact with different substances, it is possible to use sensitive graphene/SiC Schottky junctions to identify different analytes [34]. The charge transfer between heavy metals and graphene can cause a shift of the Fermi level of graphene with respect to the Dirac point, thereby changing the Schottky barrier height and, as a consequence, changing the current–voltage characteristics, which can be used as the sensor signal. The shift of the *I*–*V* curves can be calibrated to measure the concentration of heavy metals. It is important to emphasize that the key factors determining the performance of the graphene-based Schottky barrier diode as sensing platform are the quality of the interface between SiC and graphene (minimization of the density of interface states) as well as the homogeneity of the graphene thickness. Indeed, a non-uniform interface may cause the formation of a great number of surface states, a decrease in Schottky barrier height, Schottky barrier inhomogeneity and an increase in the ideality factor. As a result, different authors reported on a large variation of these parameters for such Schottky diodes based on nominally the same material [35–49]. The previously reported results of the measurements of the electrical properties of graphene/SiC Schottky diodes are listed in Table 1.

**Table 1:** A review of existing literature on fabrication method and properties of the graphene/SiC Schottky junction.

junction	growth method	thickness	Schottky barrier height [eV]	ideality factor η	ref.

graphene/*n*-Si-4H-SiC	CVD	1 ML	1.16 ± 0.16	6.5	[35]
graphene/*n*-C-4H-SiC	CVD	1 ML	1.31 ± 0.18	4.5	
graphene/*n*-4H-SiC	CVD	1 ML	0.91	1.2–5.0	[36]
graphene/*n*-4H-SiC	Si sublimation	few MLs	0.08	1.24	[37]
graphene/*n*-SiC	exfoliation	few MLs	0.28 ± 0.02	—	[38]
HOPG/*n*-SiC	van der Waals adherence of cleaved HOPG	multilayered	1.15	1.12–1.50	[39]
graphene/*n*-4H-SiC	exfoliation of HOPG	multilayered	0.8 ± 0.1	—	[40]
graphene/*n*-4H-SiC	Si sublimation	1–8 MLs	0.4 ± 0.1	—	[41]
graphene/*n*-4H-SiC	exfoliation	few MLs	0.85 ± 0.06	—	
graphene/*n*-Si-6H-SiC	CVD	1 ML	0.35 ± 0.05	—	[42]
graphene/*n*-C-4H-SiC	CVD	1 ML	0.39 ± 0.04	—	
graphene/*n*-Si-6H-SiC	thermal decomposition	2 MLs	1.15–1.45	—	[43]
graphene/*p*-4H-SiC	Si sublimation	1 ML	1.5	2	[44]
graphite/*n*-4H-SiC	solid-state graphitization	multilayered	0.3 ± 0.1	—	[45]
graphite/*p*-4H-SiC	solid-state graphitization	multilayered	2.7 ± 0.1	—	
graphene/*n*-Si-4H-SiC	thermal decomposition	few MLs	1.07 ± 0.12	1.15 ± 0.04	[46]
graphene/*n*-Si-4H-SiC	electron-beam irradiation	2 MLs	0.58	4.5	[47]
graphene/*n*-Si-4H-SiC	low-energy electron-beam irradiation	1 MLs	0.56 ± 0.05	4.5	[48]
graphene/*n*-Si-6H-SiC	thermal decomposition	2 MLs	0.9	—	[49]

From Table 1 we can see that the main parameters of the graphene/SiC junctions strongly depend on the growth method, the substrate doping, the SiC polytype and the graphene thickness. Furthermore, it was reported that the formation of ripples and ridges in graphene may be responsible for the fluctuations in the Schottky barrier height [35,42], thereby leading to increased values of the ideality factor. The most influential factors on the uniformity of the Schottky barrier height and the ideality parameter for graphene/SiC structures are the homogeneity of the graphene thickness, the quality of the grown interface (defects, pits, dislocations, surface roughness), the type of the grown interface (SiC polytypism, face polarity) and the growth conditions. Indeed, we noticed that the Schottky junctions formed by thermal decomposition are characterized by the lowest values of the ideality factor and the smallest standard deviation of the mean value of the Schottky barrier height. This is primarily due to the fact that this growth technique promotes the formation of large scale homogeneous epitaxial graphene layers. The growth of graphene on the carbon face of SiC and 6H polytype results in a barrier height that is higher than that on the Si face and 4H polytype. This can be explained by a difference in surface energy between different interfaces, which governs the growth kinetics and determines the graphene thickness. It is obvious that increasing the graphene thickness from 1 ML to multilayered graphene causes a change of the electronic properties of the carbonaceous material (energy gap, work function) and, as a consequence, the Schottky barrier height. In this case, it is not easy to control the thickness uniformity and the barrier-height distribution, which depends on the thickness. Hence, it is very important to grow homogenous epitaxial graphene monolayers and to minimize the appearance of extended defects.

Here we report on the fabrication of epitaxial graphene/Si-face-4H-SiC Schottky barrier diodes with improved barrier height uniformity, formed on uniform 1 ML graphene. Based on density functional theory (DFT) calculations and experimental findings we propose a strategy for development of a sensing platform for detection of the toxic heavy metals Cd, Hg and Pb.

## Experimental

The top-down sublimation growth process in an inductively heated furnace at 2000 °C under an argon pressure of 1 atm [50] was used to synthesize the 1 ML epitaxial graphene on *n*-type (nitrogen-doped) 4H-SiC (0001) substrates. A study of the grown samples by reflectance mapping and Raman characterization provides evidence for the formation of monolayered graphene [51]. The 1 ML coverage is found to be about 99%, thereby implying the high uniformity of the graphene thickness.

**Figure 1:**
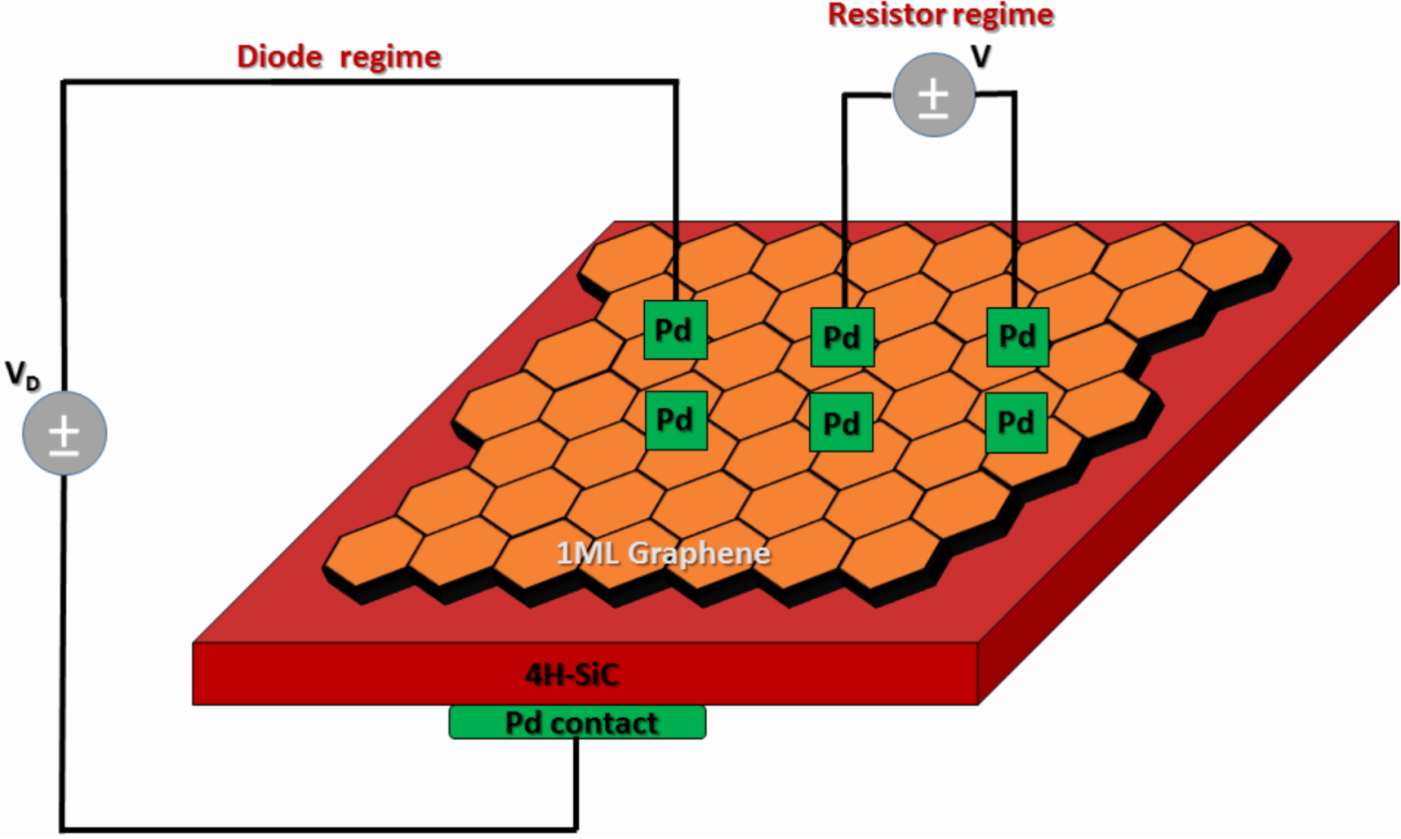
Sketch of the graphene/4H-SiC vertical device.

To avoid the necessity to etch parts of the graphene coverage in order to form the ohmic contacts to SiC, we did not utilize the lateral Schottky diode structure and mainly focused on designing vertical devices. It might be expected that the vertical structure has some advantages over the lateral device, since it offers a simpler design and a higher breakdown voltage (because of larger area efficiency). Palladium contacts (110 nm) to graphene were formed on the top side of the structure by a conventional thermal evaporation technique. Electrodes were patterned into 1 × 1 mm square shapes by the use of a shadow mask. For comparison to the diodes with Pd contacts, we have fabricated several diodes using a layer of conducting silver glue deposited on the Pd contact. As a back ohmic contact to SiC a Pd metallic electrode was also used. Figure 1 is a schematic illustration of the Pd/graphene/4H-SiC/Pd vertical device. The current-voltage (*I*–*V*) characteristics of the fabricated devices were measured and analyzed in order to extract the Schottky barrier height value, ideality factor, saturation current and series resistance. In addition, we investigated the electrical properties of the fabricated structure in the simple-resistor regime. In other words, we measured the current–voltage characteristics of the graphene resistor between two Pd metallic contacts. In fact, the detection of heavy metals can also be realized in the resistor regime, since a change in conductivity of the graphene channel after metal adsorption is expected. *I*–*V* characterization was performed in an electrical probe station (Karl Süss, Germany).

## Theoretical approach

### Analysis of diode characteristics

Assuming that the electron transport through the graphene/4H-SiC vertical structure is dominated by thermionic emission, one can write the following Richardson relation between the applied voltage and the current [52]:

[1]
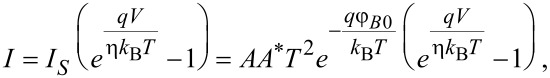


where *I*_S_ is the zero-bias saturation current, *A* is the electrically active diode area, A* is the effective Richardson constant, which is equal to 146 A·cm^−2^·K^−2^ for *n*-type 4H-SiC [46], *q* is the elementary charge, *k*_B_ is the Boltzman constant, *T* is the absolute temperature, *V* is the applied voltage, φ_B0_ is the zero-bias Schottky barrier height and η is the ideality factor. To define directly the saturation current and ideality factor from experimental *I*–*V* curves, it is convenient to re-write Equation 1 in semi-logarithmic form [52]:

[2]
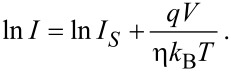


Once the semi-log plot is constructed, the slope and the *y*-axis intercept of the straight line can be used to determine ideality factor and saturation current, respectively. Knowing the value of the saturation current one can easily estimate the zero-bias Schottky barrier height [52]:

[3]
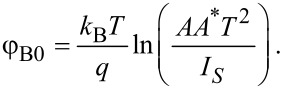


In order to obtain the series resistance *R*_S_, the Richardson equation must be written as [52]:

[4]
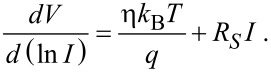


The slope of the straight line fitting this plot can be used to determine the series resistance.

The practical realization of sensors based on a graphene/SiC Schottky junction for the detection of heavy metals requires a deep understanding of how heavy metals (Cd, Pb and Hg) behave on the graphene surface and how they modify the graphene properties, such as Fermi level, work function, density of states, C–С bond lengths. Once we know the influence of heavy metals on the work function of graphene, we can predict the values of the Schottky barrier height and output characteristics of the devices (for example current–voltage characteristics). The shift of these curves can be an indicator of the presence of heavy metals. Since each of the heavy metals can be expected to behave in a unique manner on the graphene surface, e.g., different response kinetics, different shape of the response curve, careful data analysis could make it possible to draw conclusions about the selectivity of the graphene-based sensors towards different heavy metals.

### DFT analysis and computational details

An important step is to study the interaction between heavy metals and the graphene surface by DFT calculations. Furthermore, in order to establish the most favorable (from the energetic and thermodynamic point of view) geometrical configuration of incoming adsorbates, we consider the adsorption of Cd, Hg and Pb at different high-symmetry positions on the graphene surface, such as on-top site, hollow site and bridge site. For simulation of graphene, we used a graphene model of 30 carbon atoms with edges terminated by 14 hydrogen atoms (3 × 3 C_30_H_14_ cluster). To study the concentration dependence we also used the 2 × 2 (C_16_H_10_) cluster. It should be pointed out that the geometry optimization of interacting systems (graphene supercell–heavy metals) was done at the Becke3LYP level of density functional theory with a 6-31G basis set on carbon and a basis set of Stuttgart-Dresden SDD effective core potentials [53] on Cd, Hg and Pb atoms. DFT calculations of small graphene clusters and geometry optimization are performed using the default convergence criteria in the G09 package [54].

The binding energy of interacting systems were calculated based on the following relationship:

[5]
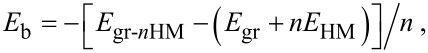


where *E*_gr-_*_n_*_HM_ is the total energy of the interacting graphene–heavy metal system, *E*_gr_ is the total energy of the isolated graphene flake, *E*_HM_ is the total energy of an individual heavy metal atom or ion and *n* is the total number of interacting heavy metal atoms or ions. To avoid the basis set superposition error (BSSE), the binding energies were calculated by means of counterpoise method [55].

The most important parameter that was extracted from our calculation, the work function of graphene before and after interaction with heavy metals, was calculated by using the following equation [56]:

[6]
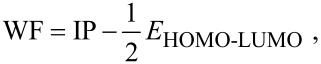


where *IP* is the ionization potential of the graphene supercell and *E*_HOMO-LUMO_ is the energy difference between the highest occupied molecular orbital (HOMO) and the lowest unoccupied molecular orbital (LUMO). It should be mentioned that in reality Equation 6 is more complex, since the effect of buffer layer, doping, functional groups (hydroxyl, carboxyl and epoxy groups) on the electronic properties of graphene cannot be ignored. The ionization potential was estimated as the energy difference between the total energy of the neutral graphene cluster and the total energy of a cation of the same cluster with +1 charge. Knowing the work function of pristine graphene and the different interacting systems one can easily predict the value of the Schottky barrier height, which forms as a result of the interaction between graphene and the SiC substrate:

[7]
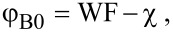


where χ is the electron affinity of silicon carbide.

To make the theoretical analysis tractable, three simplifying assumptions were used. First, we consider small H-terminated clusters as a graphene model. It is generally accepted that the small clusters behave themselves as conventional semiconductors and have an energy gap. This energy gap depends on the size of the hydrogenated graphene clusters and significantly decreases with increasing cluster size [57]. Furthermore, hydrogen atoms can contribute to the chemical interaction between sensing material and analyte, thereby leading to overestimating or underestimating some of the important parameters. It should also be mentioned that the electron–hole symmetry in doped and/or defective graphene clusters is expected to be broken and, therefore, large fluctuations in electrical parameters may occur [58].

Second, the theoretical calculations of the *I*–*V* curves of devices after applying the heavy metals were performed with consideration of the fact that the graphene distortion, which is induced by interaction of the heavy metals with graphene, can cause the appearance of an inhomogeneous graphene/SiC interface and, as a consequence, a deviation from ideal diode behavior. In this case, an ideality factor above unity might be expected. Third, we neglect the role of the SiC substrate in the chemical interaction to reduce the software execution time of Gaussian 09, although it is well known that electron charge transfer from SiC to the graphene layer is responsible for *n*-type doping in graphene. As reported earlier by Deretzis and La Magna [59], graphene–SiC substrate coupling is responsible for the appearance of midgap interface states, which can dramatically affect the electronic properties of graphene. In fact, instead of considering the interaction between neutrally charged graphene surfaces with incoming adsorbates, it would be more correct to take into account the accumulation of *n*-type charge carriers at the surface of the graphene/SiC structure to study the adsorption of metals. Nevertheless, we believe that our calculations will be useful to uncover the common trends in nature of the interaction between planar structures of carbon-containing flakes and Cd, Hg and Pb.

## Results and Discussion

### Electrical characterization of the vertical graphene/SiC Schottky diodes

**Figure 2:**
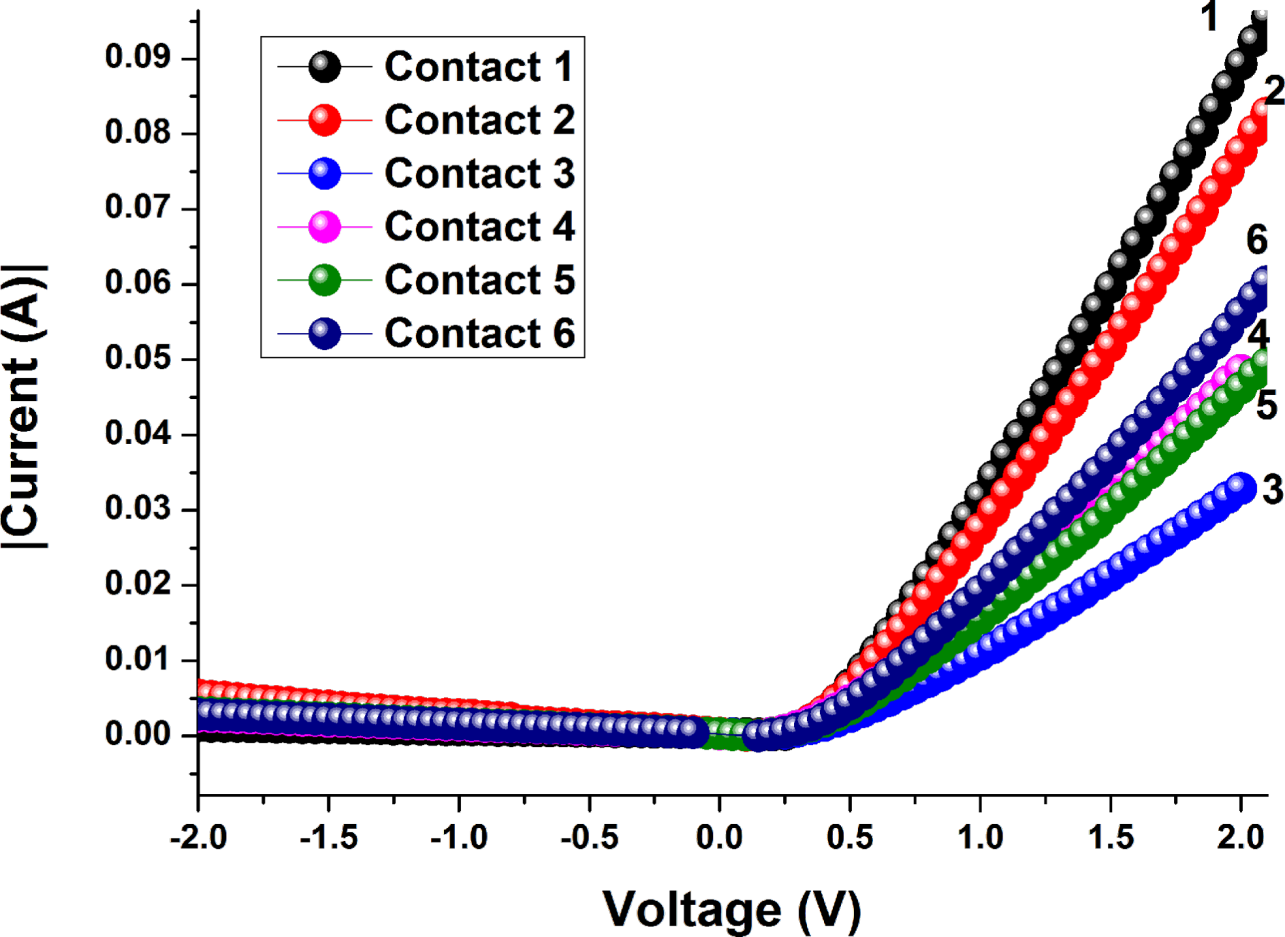
Current–voltage characteristics of the vertical graphene–4H-SiC device. The *y*-axis indicates the absolute values of current. Palladium contacts (1–6) are positioned at different places on the graphene surface*.*

As can be seen in Figure 2, the *I*–*V* characteristics of the graphene/SiC vertical devices display a rectifying behavior. The depicted curves correspond to different measurements of the same device with six Pd contacts. In fact, more than six Pd contacts were deposited on the graphene surface and we chose these six ones as representatives. It is indeed necessary to get information about the uniformity distribution of the electrical parameters (taking into account the statistical accuracy) that are suited to the fabricated graphene/SiC Schottky vertical diodes. Furthermore, the measurement of the *I*–*V* characteristics was repeated six times for each of the six contacts. It allowed us to extract the mean value of the corresponding electrical parameter and its standard deviation.

**Figure 3:**
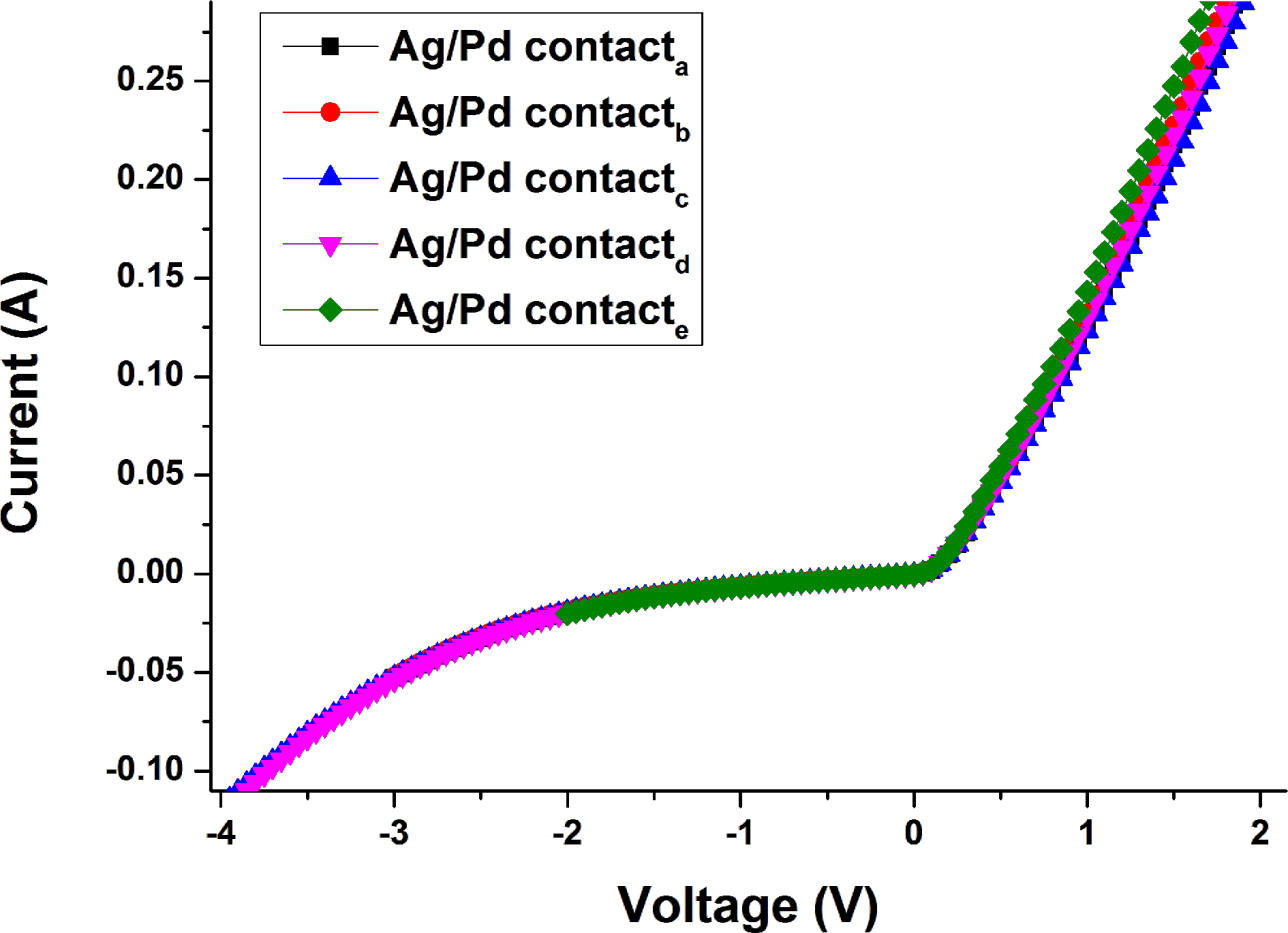
Current–voltage characteristics of the vertical device after the Pd contacts were glued with conductive silver paste.

Naturally, the question arises whether we have the Schottky barrier between graphene and 4H-SiC or between palladium and 4H-SiC. Taking into account the fact that the 1 ML coverage is approximately 99% and values for palladium Schottky contacts on *n*-type 4H–SiC (0.71–0.89 eV [60]), it is reasonable to assume that the Schottky barrier is formed mainly at the graphene/SiC interface. In order to get information about the statistical distribution of Schottky barrier height and ideality factor we carried out several additional measurements for each palladium contact. The obtained results suggest that the rectifying behavior of the graphene/SiC diode is very stable. According to the statistical distributions, the determined values of the Schottky barrier height range from 0.046 to 0.503 eV for the graphene/SiC junction, whereas the ideality factor ranges from 1.011 to 1.026. The standard deviations yield 0.013 eV and 0.0049 for both parameters, respectively. The mean values of Schottky barrier height and ideality factor of the Schottky diode are 0.4879 eV and 1.018, respectively. The extracted value of the Schottky barrier height is in good agreement with the theoretical value 0.5 eV. Indeed, within a simple approximation the barrier height at the graphene/*n*-type semiconductor interface can be determined as the difference between the work function of graphene and the electron affinity of the semiconductor. Using well-known values of 3.7 eV and 4.2 eV for the 4H-SiC electron affinity and the work function of graphene, we estimate the *n*-type barrier to be 0.5 eV. In addition, by comparing our results with reported results (Table 1), one can conclude that our sample is characterized by the smallest value of the standard deviation for the Schottky barrier height. It can be explained by assuming a high uniformity of thickness (99% according to reflectance mapping) and barrier height. The derived values of the ideality factor of a diode junction suggest that the diode exhibits almost ideal behavior, implying only a minor occurrence of interface states, generation-recombination, tunneling and even spatial inhomogeneity. The high quality of the graphene surface and the low density of defects promote also the low series resistance of the diode and the reduced leakage current. The average values of the series resistance and saturation current were found to be 34 Ω and 9.6·10^−4^ A, respectively. We also measured the *I*–*V* curves for the graphene/SiC diode after the Pd contacts were glued with conductive silver paste (Figure 3). For this case the mean value of the Schottky barrier height (0.469 ± 0.005 eV) is slightly reduced compared to the diode structure with single Pd contacts (0.488 ± 0.013 eV), while the mean values of the ideality factor and saturation current increase from 1.02 and 9.6·10^−4^ A (for a single Pd contact) to 1.03 and 1.76·10^−3^ A (for the Ag/Pd contact), respectively. Such a difference in output characteristics between vertical structures with single layer Pd contact and double layered Ag/Pd contacts can be explained by the modification of the work function of the layered contact and/or inhomogeneous surface of the contact.

The difference between Figure 2 and Figure 3 primarily originates from the quality of the contacts and unambiguously shows that the variation in the current values from the six contacts is not related to the inhomogeneity of the graphene thickness. Indeed, when Pd contacts were glued with conductive silver paste the variation in current values was smaller than that in the case of using only Pd contacts. Thus, one can conclude that controlling the quality of the contacts is an important approach to improve the performance of the Schottky barrier diode structure.

**Figure 4:**
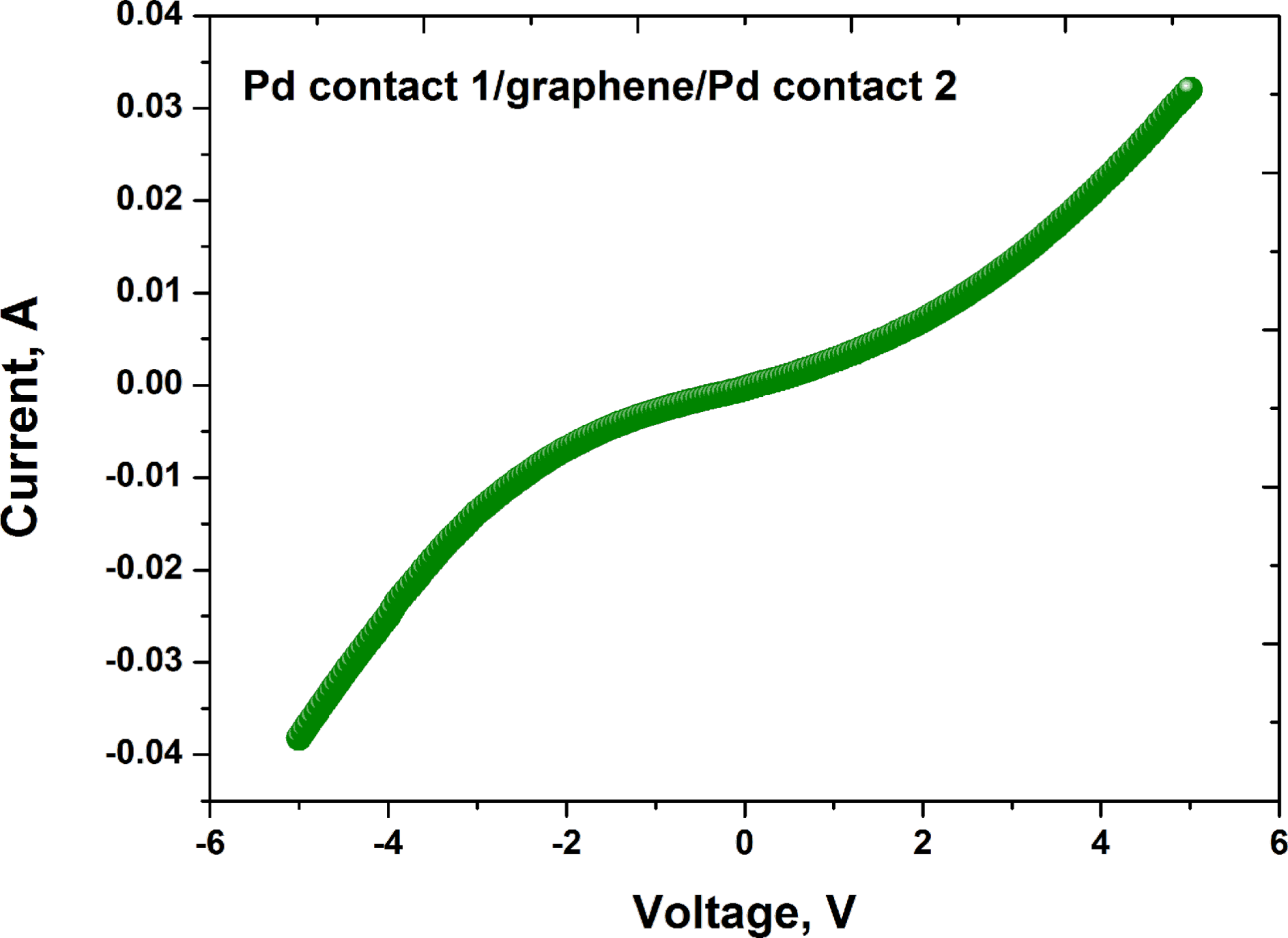
The typical *I*–*V* curve, which describes the voltage and the current flowing through the graphene channel between two palladium contacts.

Figure 4 shows the current–voltage characteristics describing the current flow through the graphene channel between two palladium contacts. As can be seen from the *I*–*V* curves presented in Figure 4, the graphene resistor demonstrates some non-linearity. There are several different reasons, which may contribute to the observed non-ohmic behavior (contact resistance, a dominant role of the recombination of carriers as compared to thermal generation, a substrate effect that provides the general mechanisms for room-temperature limitation of the carrier transport and even formation of a dual barrier diode).

### Results of DFT calculations

#### Binding strength

The second part of this paper is devoted to the discussion of the results of DFT calculations and the implementation of these results to predict the behavior of the graphene/SiC Schottky diode after interaction of graphene with heavy metals. Generally, the mechanism underlying the sensing of heavy metals is governed by the nature of the bonding between the metals and graphene. The nature of bonding is definitely depending on the geometrical position of the metal on the graphene (adsorption site) and the unique chemical properties of each metal (such as ionization potential and electron affinity). Therefore, our main goals are to illuminate the specific nature of interaction and to understand the sequence of the binding strength for heavy metals. According to our findings, the hollow site (the center of the hexagonal ring of the graphene cluster) is the most preferred site for adsorption of cadmium and mercury species, whereas the bridge site (the center of the C–C bond) is the most favorable site for the adsorption of a lead atom. The corresponding optimized distances of cadmium, mercury and lead are 3.540 Å, 3.506 Å and 2.611 Å, respectively. Thus, the maximal charge transfer might be expected between lead and graphene. To be more specific, the order of the binding strength between neutral atoms of heavy metals (located at the most favorable places) and graphene follows the sequence of Pb > Cd > Hg. It can be explained by the difference in electron affinity and ionization potential between different heavy metals. On one hand, the electron affinity of lead is approximately equal to 35.1 kJ/mol. This value is significantly higher than the electron affinity of mercury and cadmium (for these two metals electron affinity is expected to have negative or near-zero values). On the other hand, the ionization potential of the lead has the smallest value (7.416 eV) among all considered heavy metals (the ionization potentials of mercury and cadmium are 10.437 eV and 8.99 eV, respectively). Higher electron affinity indicates that an atom more easily accepts electrons (transfer of high-energy π-electrons of graphene to the metal), whereas a lower ionization potential implies stronger charge transfer from metal to graphene (metal donates an electron to graphene). In order to elucidate the nature of charge transfer between the analyte and sensing material, we also carried out a natural bond population analysis. According to our findings, the atomic charges on the heavy metals for their preferred adsorption sites were calculated to be Cd (+0.043), Hg (+0.058), and Pb (+0.284). It means that the atoms act as electron donor. At the same time, the ionic species of all considered heavy metals on the C_30_H_14_ cluster play the role of electron acceptors.

#### Electronic properties

It should be pointed out that adsorption of the heavy metals on the graphene surface drastically affects energy gap and work function of the graphene cluster. The work function of pristine C_30_H_14_ cluster is about 4.81 eV. The situation is far more complicated in the case of the adsorption of heavy metals on graphene. Upon interaction with neutral atoms of heavy metals, its energy gap and, as a consequence work function, are changed. After adsorption of Cd and Hg, the work function increases to 5.44 eV and 5.46 eV, respectively. While adding of a Pb atom to the graphene causes a decrease of the work function from 4.81 eV to 4.08 eV. Remembering from the previous discussion that a small H-terminated cluster is considered as graphene model, we would like to emphasize that the predicted value of the work function is not quantitatively exact and differs from the well-known literature data for graphene (ca. 4.2 eV). However, trends of the work function of graphene interacting with adsorbates are expected to be correctly predicted.

**Figure 5:**
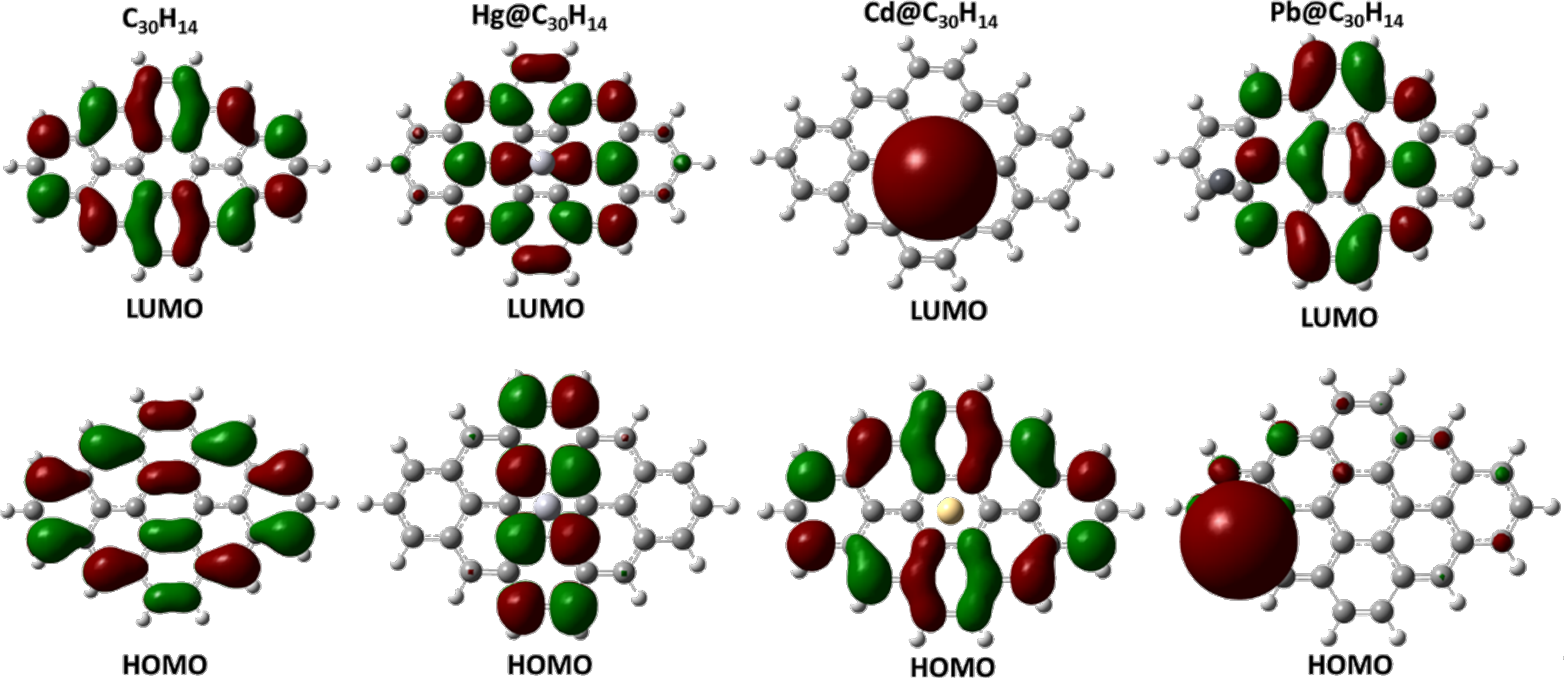
Isosurfaces of the molecular orbitals of the pristine C_30_H_14_ cluster before and after interaction with heavy metals.

Let us address the issue of electron density distributions for HOMO and LUMO as well as the energy gap of the graphene cluster before and after interaction with heavy metals. The drastic effect of the heavy metals on the electronic properties of the graphene cluster is evident from the comparison between pristine graphene and graphene interacting with Cd, Hg and Pb (see Figure 5). In particular, this effect manifests itself in a modification of the electron density distributions for HOMO and LUMO and, therefore, the HOMO–LUMO gap. In the case of pristine graphene, LUMO and HOMO are delocalized over the entire surface of the 3 × 3 graphene cluster. As can be seen from Figure 5, there is no hybridizing of the orbitals of a mercury atom and the graphene cluster (the electron distribution of the HOMO is mainly centered at the central part of the graphene cluster). Meanwhile, LUMO orbitals of Cd@C_30_H_14_ are strongly localized at the cadmium atom, whereas the HOMO is mainly distributed on the graphene sheet. Due to the chemical nature of the interaction between the graphene and lead atom, hybridization of the molecular orbitals has occurred and thus the HOMO is shared by lead adsorbate and graphene sheet. On the other hand, the LUMO of Pb@C_30_H_14_ is delocalized over the graphene sheet. In principle, such features of electron density distribution depending on the interaction of the C_30_H_14_ with heavy metals are correlated with the values of the HOMO–LUMO gap. The original HOMO–LUMO gap for a pristine graphene-like cluster estimated from the DFT calculations is approximately 2.230 eV. To our surprise, the interaction of graphene with cadmium and mercury atoms does not influence significantly the energy gap of the graphene cluster (a HOMO–LUMO value of 2.232 eV is refined in both cases). Meanwhile, a drastic change in molecular orbitals and HOMO–LUMO gap compared to the pristine graphene cluster has occurred after adsorption of Pb on the graphene surface. An energy gap of 0.772 eV was calculated for the Pb@C_30_H_14_ cluster. The order of the HOMO–LUMO gap follows the sequence of Cd@C_30_H_14_ ≈ Hg@C_30_H_14_ ≈ C_30_H_14_ > Pb@C_30_H_14_. This trend is accompanied by changes in the degree of hybridization. Indeed, the smallest value of the HOMO–LUMO gap (ca. 0.772 eV) is observed for the Pb@C_30_H_14_ cluster because of hybridization of the HOMO orbitals. Based on the derived parameters we estimate that the Schottky barrier height in the case of pristine graphene, before and after interaction with heavy metals, is connected with silicon carbide into one system. According to our estimations, the Schottky barrier height reaches a value of 1.11 eV for the pristine graphene/SiC junction and increases to values of 1.76 and 1.74 eV for Hg@C_30_H_14_/SiC and Cd@C_30_H_14_/SiC structures, respectively. At the same time, the barrier height of the junction after applying the Pb atom attains a value of 0.384 eV.

#### Dependence of the sensing parameters on the concentration of heavy metals

Because of the general tendency of some metals to cluster it is very important to investigate the dependence of the sensing parameters on the concentration of the heavy metals, since these dependences may provide useful information concerning the detection limit. With this aim, we simulated the adsorption processes on a 2 × 2 graphene flake. At the first stage, one heavy metal atom was placed on the graphene surface. We then optimized the resulting structure. At the second stage, we added a second heavy metal atom to this relaxed structure and predicted the most stable geometry. The same procedure was performed to simulate the adsorption of the third and fourth heavy metal atoms. By doing so, the role of metal–metal interaction in adsorption processes involving heavy metals on graphene can be elucidated.

**Figure 6:**
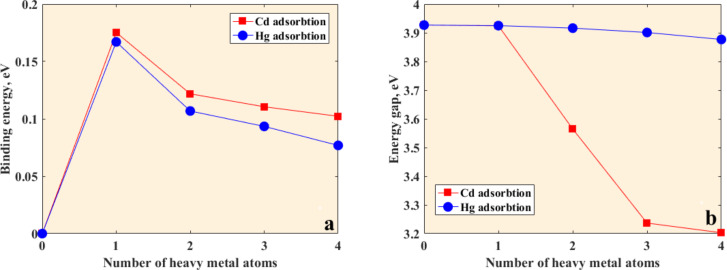
(a) Dependence of the binding energies of Cd and Hg on the number of heavy metal atoms on graphene flake. (b) Dependence of the HOMO–LUMO gap of graphene clusters interacting with Cd and Hg on the number of heavy metal atoms.

**Figure 7:**
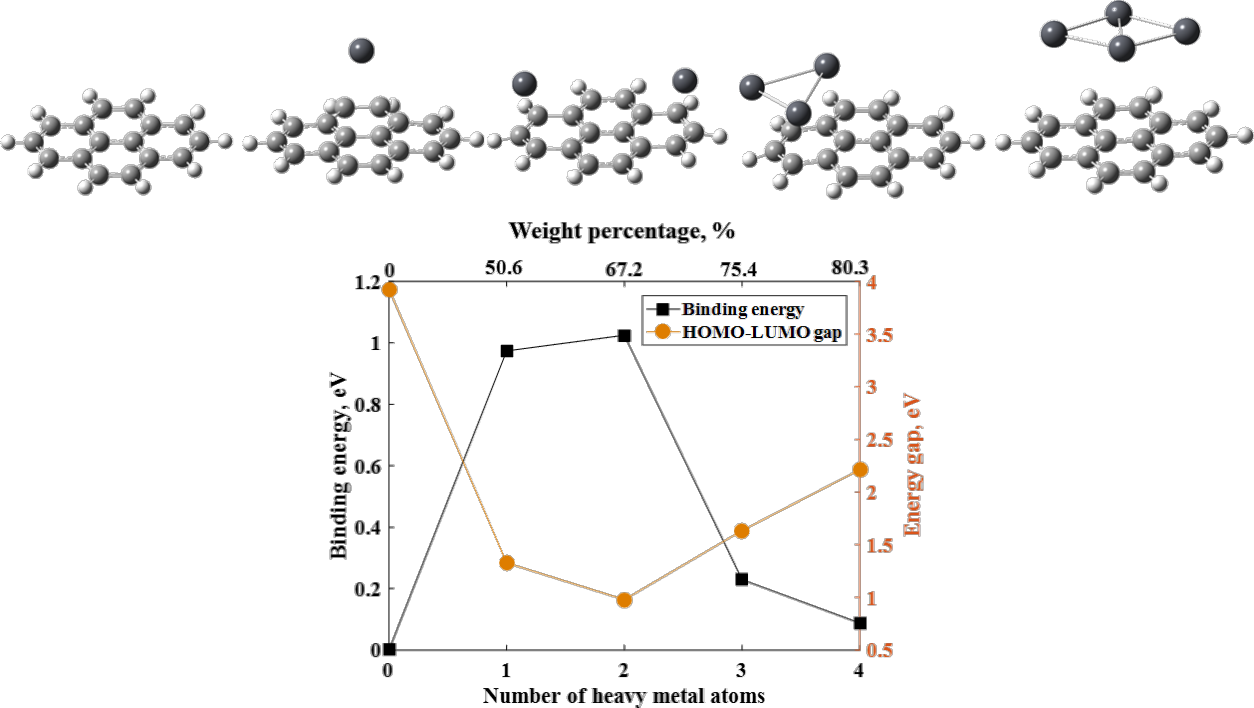
Dependence of the binding energy and HOMO-LUMO gap on number of Pb atoms. The top X axis represents the concentration of heavy metals atoms in weight percentage. The percent composition of a heavy metal atom in a system was calculated as a relationship between the total atomic weight of the Pb atoms and total weight of entire interacting systems (including graphene cluster and Pb atoms). Topmost panel shows the results of optimization obtained for different cases of adsorption of Pb atoms by the graphene surface.

Figure 6 and Figure 7 show the dependences of the binding energies of heavy metal atoms adsorbed on graphene on the number of atoms. The binding energies of isolated neutral Cd and Hg atoms on the graphene flake are 175 meV and 167 meV, respectively, but decrease upon increasing the number of adsorbed atoms (four atoms) to 102 meV and 76 meV, respectively. This is because of the aggregation of the neutral Cd and Hg atoms on the graphene surface. That is, the metal–metal attraction, which is the main driving force for metal aggregation, is stronger than the interaction between the metal atoms and the graphene flake. Increasing the number of Pb atoms results in a dramatic decrease in the binding energy, from 0.973 eV for one Pb atom to 0.088 eV for four Pb atoms. It is important to note that the presence of two Pb atoms on the graphene surface is accompanied by the strongest interaction between lead and graphene. It is evidenced by the highest binding energy. In other words, there is a critical concentration of the lead atoms at which the chemical interaction between Pb species and graphene is more energetically favorable than the formation of Pb metallic clusters.

**Figure 8:**
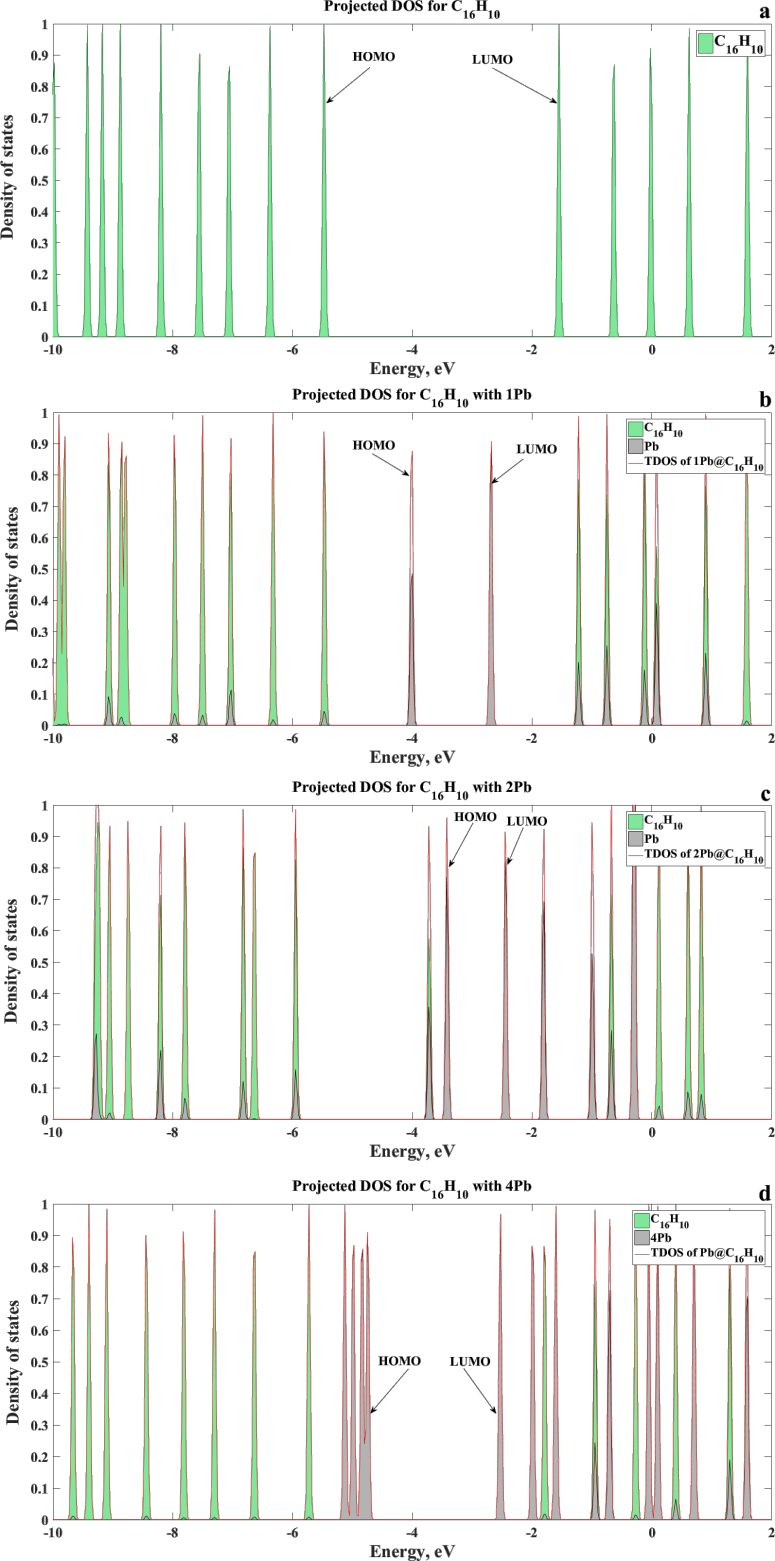
Total and projected DOS (PDOS) for graphene flakes atoms of the heavy metals: C_16_H_10_ cluster without Pb (a) C_16_H_10_ with 1 Pb (b), C_16_H_10_ with 2 Pb (c) and C_16_H_10_ with 4 Pb (d).

Further increase of the lead concentration causes a decrease in the degree of chemical interaction between Pb and graphene. This can be explained by the fact that the Pb–Pb attraction plays a more important role in binding than the interaction between Pb and graphene. Thus, the Pb clusters consisting of three and four Pb atoms are much more stable than the isolated Pb atoms on the graphene flake. The electronic gap of the graphene flake is also dependent on the amount of atoms of heavy metals (Figure 6b and Figure 7). From Figure 6b we can see the weak sensitivity of the HOMO–LUMO gap of the graphene system to the amount of Hg atoms and a stronger sensitivity on the number of Pb and Cd atoms. In the case of Pb adsorption, the energy gap dependence follows the binding energy dependence on the amount of Pb atoms. This finding is substantiated by the projected density of states in Figure 8. The Pb-related orbitals and their contribution to the states below the bottom of the conduction band and top of the valence band initially increase with increasing number of Pb atoms, and strong hybridization with C_16_H_10_-related molecular orbitals occurs. As can be seen from Figure 7 and Figure 8d, the presence of four atoms on the graphene surface significantly weakens the chemical interaction and charge transfer between them. It is evidenced also by the increase in binding height (see the optimized interacting systems illustrated by inserts in Figure 7). An observable broadening of the molecular level of Pb at −5 eV suggests significant Pb–Pb interaction, which in turn decreases the binding energy. As a result, the adsorption of the Pb cluster containing four Pb atoms by the graphene surface becomes weak.

#### Implementation of DFT results towards practical applications

**Figure 9:**
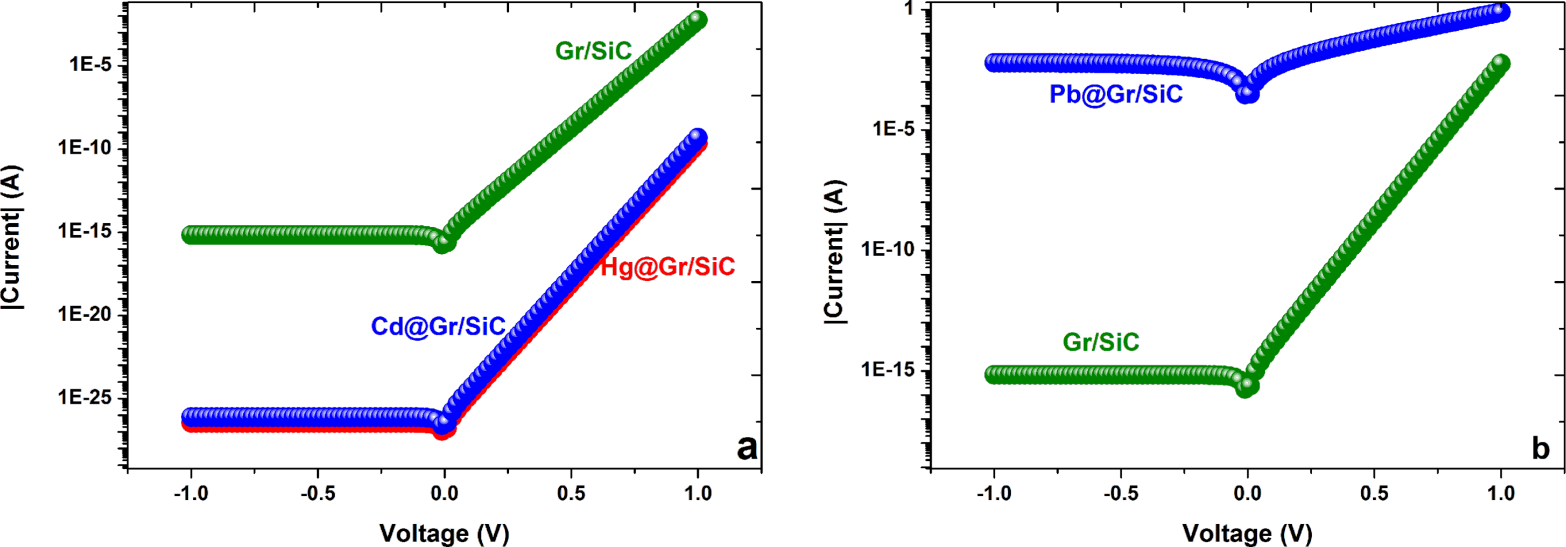
Calculated current–voltage characteristics of the graphene/SiC junction before and after interaction with Cd and Hg (a) and with Pb (b).

The calculated current–voltage characteristics of our device before and after interaction with heavy metals are shown in Figure 9. From Figure 9a, after adding the cadmium and mercury atoms, the current evidently decreases in the whole voltage region in comparison with the initial current through the pristine graphene/SiC junction. The main reason for this observable reduction is the increase in Schottky barrier height (as was discussed before). However, when interacting with lead atoms, it can be clearly seen that the current through the Schottky junction is remarkably enhanced (as shown in Figure 9b). In fact, by modifying the type of incoming adsorbates one can alter the Schottky barrier height and thus the rectifying behavior of the diode and, hence, the *I*–*V* characteristics shift.

**Figure 10:**
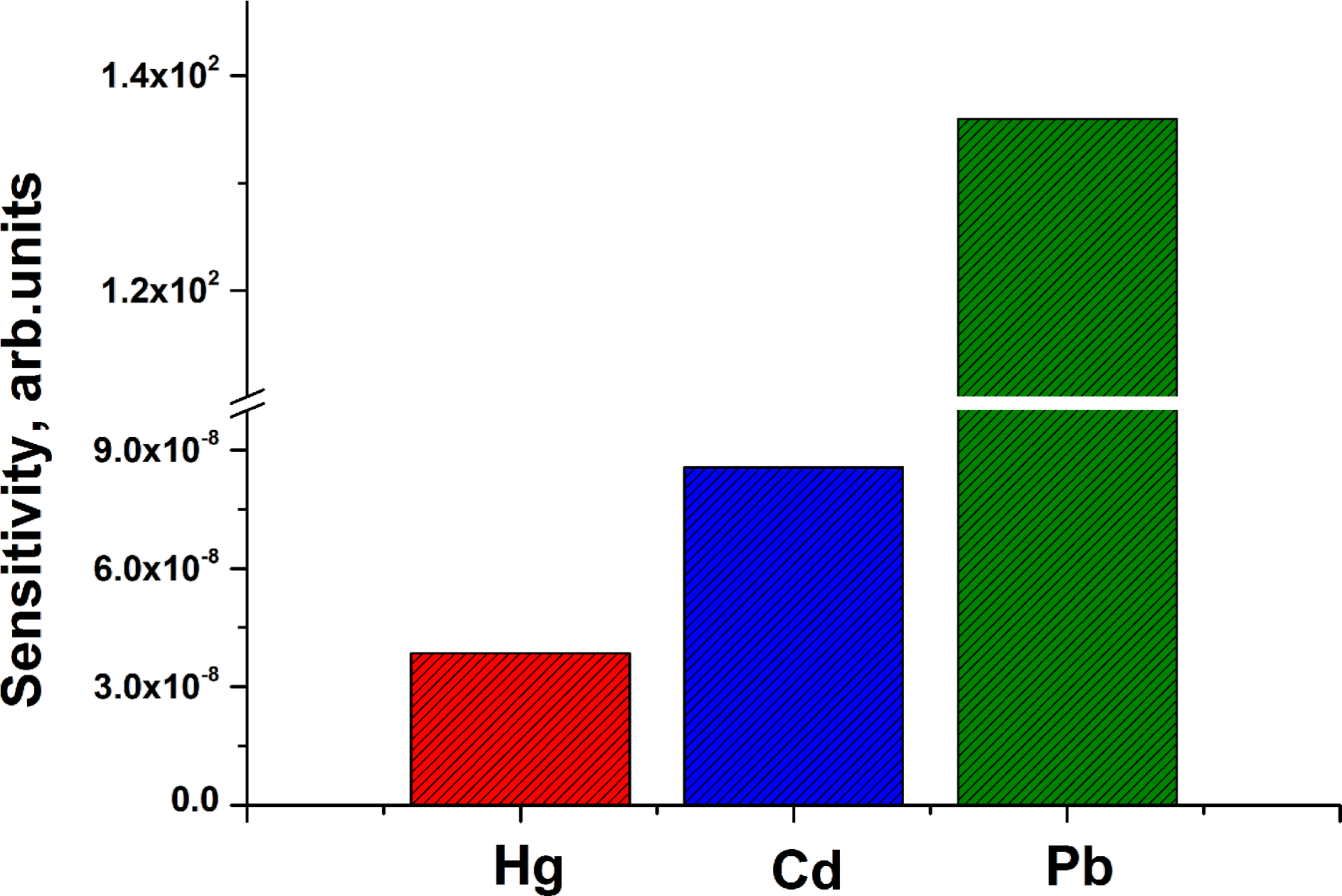
Histogram plot of graphene/SiC device sensitivity (at fixed voltage of 1 V) towards different heavy metals.

The sensitivity of the proposed device was determined from the normalized change in the initial current at a fixed voltage of 1 V. The current, following stabilization after adding heavy metals, changed depending on the type of heavy metals. A histogram of the sensitivity of the graphene/SiC structure to different heavy metals is presented in Figure 10. One can see that the selectivity of the graphene/SiC toward Pb atoms is expected to be the highest compared to the selectivity toward cadmium and mercury (Figure 10). It can be explained by the fact that the graphene has a greater binding affinity for lead atoms than for other heavy metal atoms.

**Figure 11:**
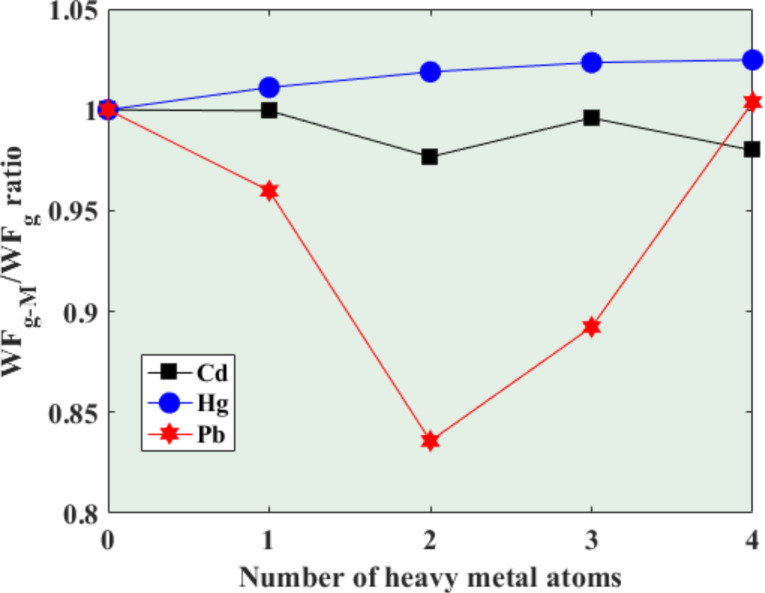
Dependence of the sensitive parameter (ratio of work functions of graphene before and after interaction with heavy metals) on the number of heavy metal atoms.

Because of the high sensitivity of the work function of graphene to adsorbates (in our case, heavy metal atoms), as the sensitivity criterion we choose the ratio between the work function of the graphene flake after interaction with heavy metals and the work function of the pristine graphene cluster. In fact, this index is proportional to the ratio between the initial current and the current following stabilization after adding the heavy metals. Therefore, this parameter can be effectively used to predict the sensitive characteristics of the proposed Schottky-based sensing platform. As shown in Figure 11, the value of this ratio is very sensitive to the change in the Pb concentration and decreases by increasing the Pb concentration up to 67.2 wt %, where it reaches a minimum and then it increases upon increasing the Pb concentration further. The dependence of the ratio on Cd and Hg concentration is very weak, therefore no significant change in current compared to the pristine graphene cluster is expected.

It is necessary to emphasize that the presence of the substrate can modify the interaction between the heavy metals and graphene. Thus, a response of a graphene system to a change from an equilibrium state can differ from theoretically predicted behavior. This difference can be related to the change in the absolute value of adsorption energy. At the same time, we believe that the general trend in adsorption of heavy metals by graphene surface will not be changed. Another important aspect is the recovery time. According to the transition state theory [61], the recovery time may be written as follows:

[8]
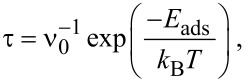


where *T* is the temperature, *k*_B_ is Boltzmann’s constant and ν_0_ is the frequency of the desorption event, and *E*_ads_ is the adsorption energy. An increase in adsorption energy will cause an increased recovery time. In fact, in the case of strong adsorption, the recovery time will be too long for the sensor signal to return to its initial value in a reasonable time. This is not applicable for real-time sensors. Our findings suggest that the recovery time of the sensor for Pb detection will be longer than that of the sensors for determination of Hg and Cd. This is mainly associated with the difference in the binding energy of different heavy metals with graphene. Anyway, a trade-off must be reached between the adsorption energy on one side and the recovery time on the other side.

The graphene/SiC interface (working in resistor regime) can also function as an effective sensor for the detection of the heavy metals. The key issue is that the heavy metal adsorption leads to band-structure reorganization and changing of the energy gap of the graphene clusters (as was discussed before). Bandgap opening may also be expected in epitaxial monolayer graphene on SiC due to the interaction with heavy metals. It is generally accepted that for many materials the fundamental relationship between the energy gap *E*_g_ and conductivity σ is valid [62]:

[9]
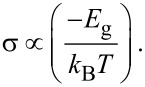


It is reasonable to assume that the decrease of the energy band after the adsorption event induces a change in the conductivity. Since the Pb adsorption leads to a decrease in the HOMO–LUMO gap of the graphene cluster, the electrical conductivity of Pb@graphene is enhanced in comparison with the pristine graphene flake and Hg/Cd@graphene and a better sensitivity towards Pb is expected.

## Conclusion

The results presented herein exhibit the possibility to use a vertical graphene/SiC junction as a sensing platform for the simultaneous detection of toxic heavy metals such as cadmium, mercury and lead. Our investigations on the graphene/SiC structure have shown that the high-temperature thermal decomposition of the Si-face 4H-SiC substrate in argon atmosphere is a good strategy to grow monolayered epitaxial graphene with high thickness uniformity and low fluctuations in barrier heights. As a result of the presence of a homogeneous interface between graphene and SiC, analysis of the current–voltage characteristics by thermionic emission approach yields a Schottky barrier height of 0.488 eV ± 0.013 eV and an ideality factor of 1.0180 ± 0.0049. These values are in good agreement with theory and suggest that the fabricated graphene/SiC Schottky diodes are characterized by stable rectifying behavior and a high degree of barrier height homogeneity. With first-principles density functional theory calculations, we demonstrated the features of the interaction of graphene with cadmium, mercury and lead atoms and the strong sensitivity of the electronic properties of pristine graphene (such as energy gap, binding energy, ionization potential and work function) to incoming adsorbates. The binding strength order predicted by DFT follows the sequence Pb > Cd > Hg. The maximal charge transfer is expected between Pb and graphene and implies the largest change in Schottky barrier height and current flowing through the barrier. For this reason, the proposed sensing platform was characterized by high selectivity towards lead atoms. It was found that an increased concentration of heavy metals leads to a weakening of the chemical interaction with graphene due to enhanced metal–metal attraction. Pb atoms tend to form more stable metallic clusters. The proposed sensing platform can selectively detect Pb atoms in the concentration range up to 67.2 wt % (2 Pb atoms per 16 carbon atoms) with a high sensitivity. Our findings expectedly offer a new reliable strategy to develop portable and real-time environmental sensors based on uniform self-organized graphene/SiC Schottky diodes.
